# Cognitive profile in prodromal dementia with Lewy bodies

**DOI:** 10.1186/s13195-017-0242-1

**Published:** 2017-03-16

**Authors:** Jennifer Kemp, Nathalie Philippi, Clélie Phillipps, Catherine Demuynck, Timothée Albasser, Catherine Martin-Hunyadi, Catherine Schmidt-Mutter, Benjamin Cretin, Frédéric Blanc

**Affiliations:** 1 Neuropsychology Unit, Neurology Department, University Hospitals of Strasbourg, Strasbourg, France; 2 Geriatrics Department, University Hospitals of Strasbourg, Geriatric Day Hospital, Strasbourg, France; 3 CMRR (Memory Resources and Research Center), University Hospitals of Strasbourg, Strasbourg, France; 4 Team IMIS/Neurocrypto, ICube Laboratory UMR 7357 and FMTS (Fédération de Médecine Translationnelle de Strasbourg), University Hospitals of Strasbourg and CNRS, Strasbourg, France; 5 INSERM Centre d’Investigation Clinique-1434, University Hospitals of Strasbourg, Strasbourg, France

**Keywords:** Dementia with Lewy bodies, Cognition, Cognitive profile, Prodromal, Mild cognitive impairment

## Abstract

**Background:**

Cortical and subcortical cognitive impairments have been found in dementia with Lewy bodies (DLB). Roughly, they comprise visuoconstructive and executive dysfunction, whereas memory would remain relatively spared. However, the cognitive profile of patients with prodromal DLB remains poorly illustrated to date.

**Methods:**

We included 37 patients with prodromal DLB (age 67.2 ± 8.6 years, 18 men, Mini Mental State Examination [MMSE] score 27.4 ± 2) and 29 healthy control subjects (HCs; age 68.8 ± 7.9 years, 15 men, MMSE score 29.0 ± 0.9). They were presented with an extensive neuropsychological test battery to assess memory; speed of processing; executive function; visuoperceptual, visuospatial and visuoconstructive abilities; language; and social cognition.

**Results:**

Compared with HCs, patients had lower scores on a visual recognition memory test (Delayed Matching to Sample-48 items; *p* ≤ 0.021) and lower free recall (all *p* ≤ 0.035), but not total recall, performance on a verbal episodic memory test (Free and Cued Selective Reminding Test). Short-term memory (*p* = 0.042) and working memory (*p* = 0.002) scores were also lower in patients. Assessment of executive function showed no slowing but overall lower performance in patients than in HCs (all *p* ≤ 0.049), whereas assessment of instrumental function yielded mixed results. Indeed, patients had lower scores on language tests (*p* ≤ 0.022), apraxia for pantomime of tool use (*p* = 0.002) and imitation of meaningless gesture (*p* = 0.005), as well as weakened visuospatial abilities (*p* = 0.047). Visuoconstruction was also impaired in patients. However, visuoperceptual abilities did not differ between groups. Finally, theory of mind abilities were lower in patients than in HCs (*p* < 0.05), but their emotion recognition abilities were similar.

**Conclusions:**

This study presents the cognitive profile in patients with prodromal DLB. In line with the literature on DLB with dementia, our results show lower performance on tests of executive function and visuoconstruction. However, we found, from a prodromal stage of DLB, memory (free recall and visual recognition) and social cognition deficits, as well as weakened visuospatial and praxic abilities.

## Background

Dementia with Lewy bodies (DLB) is the second most common form of degenerative dementia after Alzheimer’s disease (AD), with prevalence rates of up to 5% in the elderly population and up to 30% of all dementia cases [1, 2]. A recent review showed that, on the basis of 2005 revised International Consensus Criteria for DLB [1], DLB represents about 4% of dementia cases diagnosed in the community and 7.7% in secondary care [3]. DLB involves a progressive reduction in cognitive functioning, characterised by fluctuations in cognition and alertness, visual hallucinations and parkinsonism. The presence of two or three of these core signs is sufficient for a diagnosis of probable DLB [1]. Patients with DLB are also likely to present with rapid eye movement sleep behaviour disorder (RBD) and severe neuroleptic sensitivity. Other features, which are less specific, are repeated falls, autonomic dysfunction and depression.

Research on prodromal DLB—that is, the disease is present but cognitive impairment is not sufficient to lead to functional deficits in activities of daily living [4]—is relatively recent. Several features have been described in prodromal DLB [5]. For instance, behavioural and psychiatric symptoms, such as visual hallucinations, RBD, depression, anxiety and delirium, can be present very early and prior to the onset of memory impairment in DLB [6–8]. Similarly, physical symptoms, including constipation, hyposmia and postural dizziness, have been described to appear years before memory loss in prodromal DLB [7]. Pathological studies of Lewy body disease suggest that the olfactory bulb and the peripheral autonomic nervous system, including the enteric nervous system, constitute the first sites of involvement (e.g., [9, 10]). Moreover, we recently demonstrated in a neuroimaging study that patients with prodromal DLB have thinner grey matter in the right insula, superior temporal and orbitofrontal cortices than healthy control subjects (HCs) and patients with prodromal AD [11]. Similarly, we showed that patients with prodromal DLB present with diminished grey matter volumes of bilateral insulae and right anterior cingulate cortex compared with HCs [12]. Functional imaging studies using [^18^F]-fluoro-d-glucose positron emission tomography furthermore showed that patients with prodromal DLB symptoms have occipital hypometabolism [13].

Only a few studies have examined the cognitive profile of prodromal DLB, whereas cognitive impairment has been relatively well documented in patients with moderate DLB, especially compared with patients with AD or patients with Parkinson’s disease (PD) (for reviews, see [14–16]). For instance, moderate DLB is generally associated with prominent deficits on executive function tests (e.g., [17–19]), whereas verbal episodic memory and naming abilities remain rather spared (e.g., [20]). Moreover, numerous studies have shown visuoperceptual and visuospatial impairment (e.g., [14, 21]). Finally, attention is also affected, with reduced sustained and divided attention abilities and increased attentional fluctuations (e.g., [22–24]).

Studies on cognition in prodromal DLB also have been focused on the comparison with prodromal AD or PD and have revealed that patients with prodromal DLB have more visuospatial and letter fluency deficits and less memory storage deficits [25–27]. These findings are in line with the suggestion that impairment in non-memory domains (e.g., executive function, visuospatial abilities) is more likely to progress to DLB than single-domain amnestic mild cognitive impairment (MCI) [25, 27]. Researchers in a recent study assessed cognition in mild and very mild DLB [28]. The authors found that very mild DLB was associated with impairment of attentional/executive, visuospatial, visuoconstructive and naming abilities, as well as with difficulties in retrieval of episodic memory. With the progression to mild DLB, the authors found that executive function impairment increased, resulting in reduced performance on tests of inhibition, mental flexibility and verbal initiation.

The aim of the present study was to draw a cognitive profile of patients with prodromal DLB by means of an extensive neuropsychological evaluation comprising memory, executive function, instrumental function and social cognition tests. Therefore, we compared the performance on cognitive tests of patients with prodromal DLB with normative data as well as with the performance of elderly HCs.

## Methods

### Participants, diagnosis and assessments

Thirty-seven patients with prodromal DLB and 29 HCs were enrolled in the present study. Patients were recruited from the tertiary memory clinic of Strasbourg University Hospitals, Strasbourg, France, including the neurology and geriatrics departments. HCs were recruited from among friends and relatives of the patients or from among participants attending the hospital’s clinical investigation centre. Patients with prodromal DLB were defined as patients with MCI (Petersen’s criteria [29] and McKeith’s criteria [1]) or with probable DLB criteria (i.e., two core symptoms), and this maps onto recent suggestions for potential prodromal DLB criteria [5, 30]. Preservation of independence in functional abilities was assessed in patients and HCs on the basis of four items [31] of the instrumental activities of daily living [32, 33] and the activities of daily living [34] questionnaires. Participants with two or more functional domains impaired, suggesting reduced autonomy, were not included in the present study. Exclusion criteria for all participants included history of alcohol/substance abuse, evidence suggesting alternative neurological or psychiatric explanations for symptoms/cognitive impairment (for patients) or the presence of other severe or unstable medical illness. Patients additionally underwent cerebrospinal fluid (CSF) analysis, including measurement of tau, phosphorylated tau (p-Tau) and amyloid-β (Aβ) 1–42 (INNOTEST β-amyloid_(1–42)_ enzyme-linked immunosorbent assay; Fujirebio, Gent, Belgium). Assessment of medial temporal atrophy by brain magnetic resonance imaging (MRI) was performed in patients and HCs using the standardised Scheltens scale (five categories, 0–4 scale), with 0 corresponding to no atrophy [35]. Patients with concomitant DLB and AD (i.e., meeting both McKeith’s [1] and Dubois’s [36] criteria) were also excluded. More precisely, patients with DLB and two of the following features were excluded: episodic memory (storage) impairment, hippocampal atrophy (Scheltens scale of at minimum 2/4) and CSF abnormalities (at minimum two abnormal CSF markers among p-Tau, Tau, Aβ_42_ [37]).

Concretely, when hippocampal atrophy or CSF abnormalities were observed in a patient with DLB, performance on the Rappel libre/Rappel indicé à 16 items (RL/RI-16,) was checked; a patient whose performance indicated storage impairment, testifying to the presence of two of the above-mentioned features, was excluded from analysis. However, a patient presenting solely with hippocampal atrophy, CSF abnormalities or storage impairment was not excluded if the criteria for DLB were met [1]. To assess specific cognitive domains, we used the neuropsychological tests outlined below.

#### Assessment of memory

For the assessment of memory, we used the following tests:

- *The French version of the Free and Cued Selective Reminding Test* (RL/RI-16 [38]): This verbal memory test is based on semantic cuing, which allows controlling for encoding and facilitates retrieval. Sixteen words are presented that are associated with a category cue. Participants are asked to recall the words in three successive trials, then to recognise the 16 items between 32 distractors before recalling them in a 30-minute delayed trial. Each trial includes free recall (FR) and cued recall (CR) tasks whereby the category cue is provided for the items not spontaneously recalled. The total recall (TR) score is the sum of the FR and the CR.
- *The Delayed Matching to Sample-48 items* (DMS-48 [39]): The DMS-48 consists of a visual forced-choice recognition test. After an implicit encoding phase where 48 coloured items are presented, an immediate recognition trial (set 1) and a 1-h delayed recognition trial (set 2) are proposed in which participants are asked to choose between the target and a distractor. Two different sets of distractors are used.
- *Forward and backward digit spans* [40]: These tests allow evaluation of short-term and working memory. The short-term memory span is the longest list of numbers the participant can recall in correct order immediately after presentation. Backward memory span is the longest list of numbers the participant can recall in reverse order immediately after presentation.

#### Assessment of executive function

For the assessment of executive function, we used the following tests:

- *Frontal Assessment Battery* (FAB [41]): The FAB briefly assesses six cognitive function domains sustained by the frontal lobes: conceptualisation, mental flexibility, motor programming, sensitivity to interference, inhibitory control and environmental autonomy. Three points are awarded for every perfect response (maximum score 18).
- *Trail Making Test (TMT) A and B* [42]: Both parts consist of 25 circles distributed over a sheet of paper. In TMT A, the circles are numbered 1–25, and the participant is asked to draw lines to connect the numbers in ascending order as quickly as possible. In the TMT B, the circles include both numbers (1–13) and letters (A–L). The participant has to draw lines to connect the circles in an ascending pattern as quickly as possible while alternating between the numbers and letters. The completion time and the number of errors are recorded.
- *Formal lexical evocation* [43]: The participant is asked to generate as many words as possible that start with the letter P within 2 minutes.

#### Assessment of processing speed

For the assessment of processing speed, we used the digit symbol substitution test [40]. This test involves a key in which the numbers 1–9 are each paired with a unique symbol. Below the key, the numbers 1–9 are shown in random order. The participant is allowed 120 seconds to fill in the corresponding symbol for each number.

#### Assessment of instrumental function

For the assessment of instrumental function, we used the following tests:

- An oral naming test [44] of 80 pictures (maximum score 80) and formal semantic evocation [43] were used to evaluate language. Formal semantic evocation consists of generating as many names of animals as possible within 2 minutes.
- *Rey-Osterrieth Complex Figure test* (ROCF [45]): Participants were presented with the ROCF stimulus card and asked to draw the same figure. The figure is subcategorised into 18 elements, and these are scored on the basis of their presence, completeness and correct placement (0.5, 1 or 2 points per element; maximum score 36).
- The following subtests using the Visual Object and Space Perception battery (VOSP [46]) allow the evaluation of visuoperceptual and visuospatial abilities:
  - *Screening*: The participant has to identify whether there is a degraded ‘X’ on 20 patterned sheets of paper. One point is given for each correct answer (maximum score 20).
  - *Incomplete letters*: Twenty incomplete letters are shown, and the subject is asked to name or identify them. A point is awarded for each correct answer (maximum score 20).
  - *Dot count*: The participant is asked to count how many black dots there are on a white card. There are ten cards. A point is awarded for every correct count (maximum score 10).
  - *Position discrimination*: Ten boards are presented. Each board has two squares with a black dot in the centre each. In one of the squares, the point is exactly in the centre, whereas the other point is slightly off-centre. The participant is asked to identify the square in which the black spot is located exactly in the centre. The number of correct answers is recorded (maximum score 10).
  - ‘*Number location*’: Ten boards are presented in this test. Each board has two squares arranged one above the other. The top square contains numbers arranged randomly. The bottom square contains only a black dot. The participant is asked to identify which number corresponds to the black dot. Each correct identification earns 1 point (maximum score 10).
  - *Cube analysis*: Ten boards are presented. Each board features a design of solid structures. The participant is asked to identify how many solids (cubes) there are on each board. The boards are presented in increasing degree of difficulty (maximum score 10).
- Praxis is tested by the means of a brief battery [47] evaluating five symbolic gestures (scored 0 or 1 point), five pantomimes (scored 0, 1 or 2 points) and imitation of eight meaningless gestures (scored 0 or 1 point).

#### Evaluation of social cognition

For the evaluation of social cognition, we used the following tests:

- *Mini-Social Cognition & Emotional Assessment* (mini-SEA [48]) test battery: The mini-SEA comprises a facial emotion recognition test and a shortened version of the Faux Pas Recognition Test (FPRT [49]). Emotion recognition is assessed by means of 35 photographs from a series of pictures of facial affect [50]. The faces display one of the six basic emotions (i.e., happiness, sadness, disgust, fear, surprise and anger) or a neutral facial expression. After looking at each photograph, participants choose the emotion that best corresponds to their opinion of that facial expression. The maximum score, indicating best performance, is 35. The FPRT consists of ten short stories, five with and five without a faux pas. Each story has two types of questions, namely six theory of mind (ToM) questions and two control questions. The ToM questions assess the detection and understanding of faux pas and the understanding of the speaker’s and the listener’s mental states. One point is given for each correct answer on the faux pas questions, and two points are given for each correct rejection of control stories (maximum score 40). Raw scores are converted to weighted scores. Both scores are weighted out of 15, resulting in a total weighted score out of 30.
- *French version of the Reading the Mind in the Eyes (RME) test* [51]: This test evaluates the ability of an individual to determine the mental state of another individual by looking at a picture of the latter’s eyes. The task consists of 36 items showing the eye region of 36 different faces in black-and-white photographs. Each picture has four mental state terms printed below it, and the participant has to choose the word that best describes what the person in the photograph is feeling or thinking. The number of correct answers is recorded (maximum score 36).

### Data analyses

z-Scores were calculated using data derived from normal cohorts ([38–43, 46–48, 52] and Strauss and Spreen, unpublished). They are systematically adjusted for age, as well as for sex and education level when these data are available in the normal cohorts. z-Scores less than or equal to −1.65 are considered pathological. STATISTICA software (version 12.7; Statistica, Tulsa, OK, USA) was used for further statistical evaluation as required. Where appropriate, differences in demographic and clinical data were assessed using parametric (analysis of variance [ANOVA], *t* tests) and nonparametric (Kruskal-Wallis H, Mann-Whitney *U*) tests. For categorical measures, χ^2^ tests were applied. For neuropsychological tests, ANOVA for independent groups was used for the analysis of z-scores, and the nonparametric Kruskal-Wallis H test was used for the analysis of raw scores. For each test statistic, a probability value less than 0.05 was regarded as significant.

## Results

### Participants’ characteristics

Demographic data for patients and HCs are summarised in Table 1. The groups did not differ in terms of age, education, sex and handedness.

**Table 1:** Demographic and clinical characteristics of patients and healthy control subjects

Characteristic	Patients with DLB	HCs	*p* Value
*n*	37	29	
Age, years^a^	67.19 (8.64)	68.79 (7.94)	NS
Education, years^a^	11.97 (4.14)	13.18 (3.08)	NS
Sex, M/F	18/19	15/14	NS
Handedness, R/L	35/2	27/2	NS
IADL score^a,b^	3.75 (0.50)	4 (0)	0.02
ADL score^a,c^	5.89 (0.39)	6 (0)	NS
MCI single/multiple domains			
Amnestic	0/18	–	–
Non-amnestic	10/9	–	–
Parkinsonism, *n* (%)^d^			
Rigidity	28/37 (76)	0/23	<0.001
Akinesia	22/37 (59)	1/24	<0.001
Tremor at rest	10/37 (27)	1/24	0.02
Hallucinations, *n* (%)^d^	24/37 (65)	1/24	<0.001
Fluctuations, *n* (%)^d,e^	28/37 (76)	0/24	<0.001
CSF^f,g^			
Aβ_42_	902.6 (265.1, 2)	–	–
p-Tau	43.4 (12.2, 2)	–	–
Tau	306.1 (264.1, 1)	–	–
Hippocampal atrophy, 0/1/2/3/4^h^			
Left hippocampus	17/8/9/2/0	14/8/2/0/0	NS
Right hippocampus	15/10/11/0/0	11/11/2/0/0	NS

*Abbreviations: Aβ*_*42*_ Amyloid-β 42, *ADL* Activities of daily living, *CSF* Cerebrospinal fluid, *DLB* Dementia with Lewy bodies, *HC* Healthy control subjects, *IADL* Instrumental activities of daily living, *MCI* Mild cognitive impairment, *NS* Not significant, *p-Tau* Phosphorylated tau

^a^Values are mean (SD)

^b^According to [32, 33]

^c^According to [34]

^d^Data partially missing for six HCs

^e^According to the Mayo Fluctuations Questionnaire [69]

^f^Data missing for ten patients

^g^Values are mean (SD, patients with abnormal values)

^h^According to [35]; one patient with DLB and five HCs did not have magnetic resonance imaging scans

### Cognition

Neuropsychological test results (raw scores and z-scores) of patients and HCs are reported in Table 2.

**Table 2:** Neuropsychological test raw scores and z-scores of patients and healthy control subjects

		Patients with DLB (*n* = 37)		HCs (*n* = 29)		*p* Value
		Raw score^a^	z-Score^a^	Raw score^a^	z-Score^a^	
Global functioning	MMSE (score/30)	27.36 (1.97)		29.00 (0.90)		0.008
Memory						
FCSRT	IR	15.11 (1.72)	−0.17 (2.34)	15.90 (0.31)	0.81 (0.49)	NS
	FR1	7.31 (2.36)	−0.66 (1.00)	8.79 (2.09)	0.02 (0.78)	0.006
	FR2	8.28 (2.91)	−0.55 (1.18)	10.62 (2.43)	0.40 (0.91)	0.001
	FR3	9.89 (2.67)	−0.51 (1.01)	11.38 (2.50)	0.08 (0.91)	0.035
	TR1	14.22 (1.85)	−0.27 (1.12)	15.07 (1.22)	0.29 (0.62)	NS
	TR2	14.81 (2.03)	−0.35 (1.76)	15.72 (0.65)	0.49 (0.40)	NS
	TR3	15.43 (0.95)	−0.11 (1.04)	15.83 (0.38)	0.31 (0.46)	NS
	Recognition	15.79 (0.59)	0.16 (0.93)	15.89 (0.31)	0.35 (0.53)	NS
	DFR	9.97 (3.82)	−0.50 (1.69)	12.45 (1.66)	0.53 (0.68)	0.003
	DTR	15.15 (1.67)	−0.46 (2.05)	15.90 (0.31)	0.47 (0.66)	NS
DMS-48	Set 1	**44.05 (5.35)**	**−2.72 (5.71, 32.4)**	46.93 (1.28)	0.21 (0.97)	0.021
	Set 2	**44.23 (4.73)**	**−2.14 (4.05, 48.6)**	47.10 (0.94)	0.14 (0.72)	0.008
Digit span (number of digits)		5.25 (1.13)		5.92 (1.02)		0.042
Executive function						
FAB		**15.49 (2.67)**	**−2.81 (4.61, 32.4)**	17.29 (1.15)	−0.02 (1.44)	0.014
TMT A		**64.89 (34.11)**	**3.00 (4.32, 51.4)**	40.62 (11.00)	0.09 (0.90)	0.011
TMT B		**139.13 (79.20)**	**3.06 (6.45, 45.9)**	90.34 (31.56)	0.12 (1.00)	0.049
Digit span backward (number of digits)		3.61 (0.90)		4.42 (0.83)		0.002
Formal lexical evocation		17.28 (7.42)	−0.45 (1.09)	23.55 (7.11)	0.38 (1.09)	0.007
Digit symbol (standard score/19)		8.18 (3.23)		11.69 (2.65)		NS
Instrumental function						
Praxis	Symbolic gesture (score/5)	4.81 (0.46)		4.90 (0.41)		NS
	Pantomime of tool use (score/10)	9.22 (1.03)		9.83 (0.38)		0.002
	Imitation of meaningless gesture (score/8)	6.92 (1.44)		7.76 (0.69)		0.005
Language	DO80	77.61 (2.78)	0.39 (1.04)	79.62 (0.62)	0.95 (0.27)	0.022
	Formal semantic evocation	25.00 (7.72)	−0.88 (1.36)	37.38 (7.05)	0.69 (0.94)	<0.001
Visuoconstruction	ROCF	**31.44 (5.25)**	**−1.95 (3.41, 32.4)**	34.37 (1.82)	0.13 (0.68)	NS
Visuoperception (VOSP)	Screening (score/20)	19.55 (1.09)		19.96 (0.20)		NS
	Incomplete letters (score/20)	18.85 (1.66)		19.41 (0.80)		NS
	Dot counting (score/10)	9.78 (0.55)		10.00 (0.00)		NS
	Position discrimination (score/20)	19.05 (1.43)		18.73 (2.39)		NS
	Number location (score/10)	8.47 (1.78)		9.46 (0.81)		0.047
	Cube analysis (score/10)	9.47 (1.11)		9.71 (0.55)		NS
Social cognition						
Mini-SEA	FPRT	11.40 (2.39)	−1.20 (1.59)	12.73 (2.39)	−0.32 (1.11)	0.026
	Facial emotion recognition	12.12 (1.44)	−0.44 (1.31)	12.44 (1.43)	−0.15 (1.30)	NS
RME test		21.19 (4.95)	−0.19 (1.23)	23.19 (3.61)	0.35 (0.88)	0.046

*Abbreviations: MMSE* Mini Mental State Examination, *FCSRT* Free and Cued Selective Reminding Test, *IR* immediate recall, *FR* free recall, *TR* total recall, *DFR* delayed free recall, *DTR* delayed total recall, *DMS-48* Delayed Matching to Sample-48 items, *FAB* Frontal Assessment Battery, *TMT* Trail Making Test, *DO80* Oral Denomination–80 items, *ROCF* Rey-Osterrieth Complex Figure test, *VOSP* Visual Object and Space Perception battery, *Mini-SEA* Mini-Social Cognition & Emotional Assessment, *FPRT* Faux Pas Recognition Test, *RME* Reading the Mind in the Eyes, *NS* Not significant

Values are mean (SD, % of patients presenting with impairment with regard to normative data); raw scores or z-scores indicating impairment with regard to normative data are shown in boldface type

^a^Raw scores: MMSE, digit span, FAB, digit span backward, digit symbol, praxis, VOSP; z-scores: FCSRT, DMS-48, TMT, formal lexical evocation, DO80, formal semantic evocation, ROCF, mini-SEA, RME test

The HCs performed within the normal range on all tests. In patients with DLB, analyses revealed pathological scores on the DMS-48 (z-scores −2.72 and −2.14 for set 1 and set 2, respectively) for visual recognition memory, on the ROCF (z-score −1.95) for visuoconstructive abilities, and on the FAB (z-score −2.81) and the TMT (z-scores 3.00 and 3.06 for TMT A and TMT B, respectively) for executive function.

In addition to the impairment demonstrated by patients with DLB highlighted above, we found significant differences between patients and HCs on other tests assessing memory, namely in FR of the RL/RI-16, reflecting retrieval of episodic memory (*p* = 0.006, *p* = 0.001, *p* = 0.035 and *p* = 0.003, for FR1, FR2, FR3 and delayed FR, respectively) and in short-term memory (*p* = 0.042), but not for TR of the RL/RI-16. Other executive function measurements were also significantly lower in patients with DLB than in HCs (*p* = 0.002 and *p* = 0.007 for working memory and formal lexical evocation, respectively). Similarly, some instrumental functions were decreased in patients with DLB compared with HCs, with significant differences for praxis (*p* = 0.002 for pantomime of tool use and *p* = 0.005 for imitation of meaningless gesture), language (*p* = 0.022 for oral naming and *p* < 0.001 for formal semantic evocation) and visuospatial abilities when assessed with the ‘number location’ subtest of the VOSP (*p* = 0.047). Finally, patients with DLB performed significantly poorer on tests evaluating mind reading (i.e., FPRT and RME test; *p* = 0.026 and *p* = 0.046, respectively).

According to Petersen’s criteria [29], 27.03% of our patients presented with nonamnestic single-domain MCI and 24.32% with non-amnestic multiple-domain MCI. The remaining 48.65% of our patients presented with amnestic multiple-domain MCI (defined by storage impairment); none had amnestic singledomain MCI. Among the patients with amnestic multiple-domain MCI, ten were impaired exclusively on a visual memory test (i.e., DMS-48), one exclusively on a verbal memory test and seven on both visual and verbal memory tasks.

## Discussion

The aim of the present study was to highlight the cognitive profile of patients with prodromal DLB. The results reveal the presence of executive, visual memory and visuoconstructive deficits from very early stages of the disease. Indeed, in our patients with DLB, these abilities appeared to be impaired with regard to normative data. Moreover, we highlighted weaknesses in some cognitive functions in our patients with DLB in that their performance on some neuropsychological tests was significantly lower than that of the HCs.

### Visuoconstruction

Our patients had pathological scores on the copy of the ROCF. Visuoconstructive impairments have previously been found in patients with DLB at moderate and mild stages [14, 17, 27]. It has even been suggested that a reduced number of angles on the Mini Mental State Examination (MMSE) pentagon copy could be a marker of prodromal DLB, with a specificity of 91% in discrimination from AD [53]. However, DLB is also associated with visuoperceptual deficits (see [54] for a review) and praxic difficulties [55]. Hence, it has been suggested that copying impairments in patients with DLB might be linked to combined praxis and visuoperceptual disturbances [56] instead of reflecting pure visuoconstructive impairment. Our results would appear to support this hypothesis insofar as we found a weakness in visuospatial abilities and praxis. Indeed, our patients performed significantly worse than the HCs in the ‘number location’ subtest of the VOSP, in pantomime of tool use, and in imitating meaningless gestures.

Nevertheless, more frank difficulties in visuospatial abilities or difficulties in visuoperceptual abilities cannot be excluded. Indeed, the performance of our patients with DLB (and HCs) was nearly at ceiling on the screening, incomplete letters, dot counting and cube analysis subtests of the VOSP, which is likely to hide potential slight difficulties in prodromal DLB [57]. Indeed, in patients with DLB with lower MMSE scores (i.e., 19–20), significantly lower performance on these subtests than that of HCs was observed [18, 58]. Other visuoperception tests might have been more sensitive. For example, results of the silhouettes, object decision and progressive silhouettes subtests of the VOSP have been found to be impaired in patients with prodromal DLB (mean MMSE score 27.8) [57]. Moreover, although tested in patients with more advanced DLB (i.e., mean MMSE score 19), overlapping figure identification [21] or an illusory contours test [59] could have been an alternative.

### Executive function

Our results show that executive function is also compromised in early DLB, as shown by the patient group’s pathological score on the FAB. Shifting, assessed by means of the TMT B, also appeared to be impaired. However, analyses revealed that patients’ performance on the TMT A was also below the normal range. Consequently, one might question whether the impairment seen on the TMT B truly reflects shifting difficulties. Indeed, the TMT involves visual search, which is impaired in DLB [60] and consequently may account for the observed impairment on the TMT. Moreover, the average z-scores of our patients were similar in both parts of the TMT (i.e., A and B). This suggests that visual search difficulties are more likely to explain the highlighted impairment than a disturbance of shifting abilities. Indeed, the latter would have resulted in a disproportionate increase in the completion time (and consequently in the z-score) in TMT B compared with TMT A. An overall slowing could also account for the longer completion time. However, this appears more unlikely, given that patients’ performance was within the normal range and did not differ significantly from that of the HCs on the digit symbol test, which specifically assesses processing speed.

Nonetheless, as suggested previously [14], non-graphomotor tests might be more sensitive in identifying pure executive impairment in this disease. This hypothesis is supported by our findings showing that patients experienced difficulties in verbal executive tasks, namely in the formal lexical evocation task assessing verbal initiation, in the digit span backward test evaluating working memory, and in retrieval of episodic memory (see below for a discussion of the latter).

### Memory

Visual recognition memory appeared to be impaired in our patients, with pathological scores on both sets of the DMS-48, whereas verbal memory appeared to be better preserved. These findings are in line with those of other studies in which researchers found impairment in patients with DLB on the basis of the DMS-48 [61] and generally better performance of patients with DLB in verbal memory tests than on visual memory tests (e.g., [14]). However, it is possible that difficulties in cortical visual and/or attentional abilities contribute to the visual memory impairment highlighted in DLB. For instance, although our patients’ scores were pathological on both sets of the DMS-48, they were stable between set 1 and set 2. A recent study by our team [62] showed that a decrease in performance between set 1 and set 2 of the DMS-48 in patients with MCI indicates medial temporal lobe dysfunction, which is known to result in a storage (i.e., memory) deficit. In contrast, scores that remain stable between the two sets, as in our patients, are reported to be correlated with extramedial temporal lobe regions, namely the temporal and parietal cortices, cerebral areas implicated in visual identification and visual attentional processes [62].

Regarding verbal memory, we found that although patients had scores that remained within the normal range, they performed poorly on the RL/RI-16. More precisely, they had significantly lower scores than HCs on all FRs (i.e., immediate and delayed), indicating weakened retrieval abilities. These results are in line with those of Petrova et al. [27], who found significant deficits in retrieval from episodic memory in patients with very mild DLB. Hence, these findings indicate retrieval (i.e., executive) impairment rather than real verbal memory (i.e., storage) impairment in prodromal DLB. However, this should be qualified. Indeed, 8 (21.6%) of our 37 patients with DLB presented with amnestic MCI reflected by storage impairment in verbal memory. This finding is of high importance because it highlights that verbal memory impairment can be present in DLB from very early stages and it is not exclusively an indicator of prodromal AD. Finally, our patients with DLB performed poorer than HCs on the digit span test, indicating decreased short-term memory, possibly linked to attentional disturbances and fluctuations known to be present from a prodromal stage [25].

### Language

Our patients with prodromal DLB manifested poorer performance on expressive language tests. More precisely, they scored lower than HCs in verbal production (i.e., formal semantic evocation) as well as in oral naming. These results are in line with some studies (e.g., [28, 63]) but contrast with studies showing a relative preservation of naming abilities in DLB (e.g., [64]). However, when language difficulties are present, the underlying mechanisms are not fully elucidated [16]. One hypothesis is that the visuoperceptual difficulties often present in DLB (see [54] for a review) might affect patients’ picture recognition and consequently their performance in picture naming. Another hypothesis emphasises that attentional and executive deficits could contribute to difficulties in word search and retrieval [16]. Yet another hypothesis is that the observed difficulties might be linked to semantic disorders. Indeed, the presence of semantic difficulties in DLB has previously been suggested [63]. For instance, [63] found that, although patients with DLB exhibited more severe semantic deficits for pictures than for words, they had lower performance than HCs in all administered assessments of semantic memory. The analysis of two qualitative indices of fluency performance, namely switching and clustering, would have been of interest to elucidate the mechanisms underlying the verbal fluency deficits. In PD, which shares some clinical features with DLB, switching impairments are more common than clustering difficulties [65, 66], thus indicating an executive function disturbance. Moreover, our patients’ performance on the formal lexical evocation task is also significantly lower than that of the HCs. This task assesses only executive function (verbal initiation) and not semantic memory.

### Social cognition

Finally, we assessed different facets of social cognition—namely, emotion recognition, mind reading and recognition of faux pas—abilities referred to as *theory of mind*. Our results suggest that emotion recognition is not affected in prodromal DLB. In contrast, difficulties in ToM are present from a prodromal stage of the disease. Indeed, although our patients’ performance remained within the normal range according to normative data, their performance was significantly worse than HCs on the RME test and the FPRT. These findings are in line with a previous publication by our group on cognitive and affective ToM in early DLB [67]. Nevertheless, assessment of social cognition, and especially the attribution of feelings derived from pictures, implies additional cognitive functions, such as visuoperceptual and visuospatial abilities. Therefore, the observed difficulties of patients with DLB on the RME tests need to be interpreted with caution, given that visuospatial capacities appear weakened from a prodromal stage of DLB. Similarly, Aboulafia-Brakha et al. [68] recently emphasised in a review that performance on both the FPRT and the RME test was strongly correlated with executive function, which is also affected from a prodromal stage of DLB. Nevertheless, a deficiency in social cognition, and especially in affective ToM, remains likely. Indeed, when analysing performance on the different questions of the FPRT separately, it appears that patients with DLB are the most impaired on question 6 [67], which assesses emotional attribution and empathy and requires hardly any involvement of executive function.

## Conclusions

This study presents the cognitive profile of individuals with prodromal DLB. We found that impairment on the basis of tests assessing visual memory, executive function and visuoconstructive abilities was present from a very early stage of the disease. Moreover, some cognitive weaknesses were highlighted: Patients performed more poorly than HCs on tests evaluating retrieval of episodic memory, short-term and working memory, verbal initiation, praxis, language, visuospatial abilities and social cognition.

In prodromal DLB, the cognitive difficulties and weaknesses seem diffuse; yet, it appears that difficulties in cortical visual abilities and executive abilities are prominent and are likely to account at least partially for cognitive disturbances observed in neuropsychological tests assessing other cognitive functions. Further studies are needed to better understand the neural basis of these cognitive deficits in prodromal DLB. Similarly, with regard to recent findings based on imaging studies, the assessment of cognitive function underpinned by the insula would be of high interest.

## Abbreviations

AD: Alzheimer’s disease
ADL: Activities of daily living
ANOVA: Analysis of variance
Aβ_42_: Amyloid-β 42
CR: Cued recall
CSF: Cerebrospinal fluid
DFR: Delayed free recall
DLB: Dementia with Lewy bodies
DMS-48: Delayed Matching to Sample-48 items
DO80: Oral Denomination–80 items
DTR: Delayed total recall
FAB: Frontal Assessment Battery
FCSRT: Free and Cued Selective Reminding Test
FPRT: Faux Pas Recognition Test
FR: Free recall
HC: Healthy control subjects
IADL: Instrumental activities of daily living
IR: Immediate recall
MCI: Mild cognitive impairment
mini-SEA: Mini-Social Cognition & Emotional Assessment
MMSE: Mini Mental State Examination
MRI: Magnetic resonance imaging
NS: Not significant
PD: Parkinson’s disease
p-Tau: Phosphorylated tau
RBD: Rapid eye movement sleep behaviour disorder
RL/RI-16: Rappel libre/Rappel indicé à 16 items
RME: Reading the Mind in the Eyes
ROCF: Rey-Osterrieth Complex Figure test
TMT: Trail Making Test
ToM: Theory of mind
TR: Total recall
VOSP: Visual Object and Space Perception battery

## References

[1] McKeith IG, Dickson DW, Lowe J, Emre M, O’Brien JT, Feldman H (2005). Diagnosis and management of dementia with Lewy bodies: third report of the DLB Consortium. Neurology.

[2] Zaccai J, McCracken C, Brayne C (2005). A systematic review of prevalence and incidence studies of dementia with Lewy bodies. Age Ageing.

[3] Vann Jones SA, O’Brien JT (2014). The prevalence and incidence of dementia with Lewy bodies: a systematic review of population and clinical studies. Psychol Med.

[4] Boeve BF (2012). Mild cognitive impairment associated with underlying Alzheimer’s disease versus Lewy body disease. Parkinsonism Relat Disord..

[5] Donaghy PC, McKeith IG (2014). The clinical characteristics of dementia with Lewy bodies and a consideration of prodromal diagnosis. Alzheimers Res Ther.

[6] Auning E, Rongve A, Fladby T, Booij J, Hortobagyi T, Siepel FJ (2011). Early and presenting symptoms of dementia with Lewy bodies. Dement Geriatr Cogn Disord.

[7] Chiba Y, Fujishiro H, Iseki E, Ota K, Kasanuki K, Hirayasu Y (2012). Retrospective survey of prodromal symptoms in dementia with Lewy bodies: comparison with Alzheimer’s disease. Dement Geriatr Cogn Disord.

[8] Fujishiro H, Iseki E, Nakamura S, Kasanuki K, Chiba Y, Ota K (2013). Dementia with Lewy bodies: early diagnostic challenges. Psychogeriatrics.

[9] Beach TG, White CL, Hladik CL, Sabbagh MN, Connor DJ, Shill HA (2009). Olfactory bulb α-synucleinopathy has high specificity and sensitivity for Lewy body disorders. Acta Neuropathol.

[10] Beach TG, Adler CH, Sue LI, Vedders L, Lue L, White CL (2010). Multi-organ distribution of phosphorylated α-synuclein histopathology in subjects with Lewy body disorders. Acta Neuropathol.

[11] Blanc F, Colloby SJ, Philippi N, de Petigny X, Jung B, Demuynck C (2015). Cortical thickness in dementia with Lewy bodies and Alzheimer’s disease: a comparison of prodromal and dementia stages. PLoS One.

[12] Blanc F, Colloby SJ, Cretin B, Loureiro de Sousa P, Demuynck C, O’Brien JT (2016). Grey matter atrophy in prodromal stage of dementia with Lewy bodies and Alzheimer’s disease. Alzheimers Res Ther.

[13] Fujishiro H, Iseki E, Murayama N, Yamamoto R, Higashi S, Kasanuki K (2010). Diffuse occipital hypometabolism on [^18^F]-FDG PET scans in patients with idiopathic REM sleep behavior disorder: prodromal dementia with Lewy bodies?. Psychogeriatrics.

[14] Noe E, Marder K, Bell KL, Jacobs DM, Manly JJ, Stern Y (2004). Comparison of dementia with Lewy bodies to Alzheimer’s disease and Parkinson’s disease with dementia. Mov Disord.

[15] Metzler-Baddeley C (2007). A review of cognitive impairments in dementia with Lewy bodies relative to Alzheimer’s disease and Parkinson’s disease with dementia. Cortex.

[16] Troster AI (2008). Neuropsychological characteristics of dementia with Lewy bodies and Parkinson’s disease with dementia: differentiation, early detection, and implications for “mild cognitive impairment” and biomarkers. Neuropsychol Rev.

[17] Simard M, van Reekum R, Cohen T (2000). A review of the cognitive and behavioral symptoms in dementia with Lewy bodies. J Neuropsychiatry Clin Neurosci.

[18] Calderon J, Perry RJ, Erzinclioglu SW, Berrios GE, Dening TR, Hodges JR (2001). Perception, attention, and working memory are disproportionately impaired in dementia with Lewy bodies compared with Alzheimer’s disease. J Neurol Neurosurg Psychiatry.

[19] Collerton D, Burn D, McKeith IG, O’Brien JT (2003). Systematic review and meta-analysis show that dementia with Lewy bodies is a visual-perceptual and attentional-executive dementia. Dement Geriatr Cogn Disord.

[20] Aarsland D, Litvan I, Salmon D, Galasko D, Wentzel-Larsen T, Larsen JP (2003). Performance on the Dementia Rating Scale in Parkinson’s disease with dementia and dementia with Lewy bodies: comparison with progressive supranuclear palsy and Alzheimer’s disease. J Neurol Neurosurg Psychiatry.

[21] Mori E (2000). Dementia with Lewy bodies [in Japanese]. Nihon Ronen Igakkai Zasshi.

[22] Hansen L, Salmon D, Galasko D, Masliah E, Katzman R, DeTeresa R (1990). The Lewy body variant of Alzheimer’s disease: a clinical and pathologic entity. Neurology.

[23] Ayre G, Ballard C, Pincock C, McKeith IG, Sahgal A, Wesnes K (1998). Double dissociation between dementia with Lewy bodies and Alzheimer’s disease on tests of attentional and mnemonic function: the role of the basal forebrain. J Psychopharmacol..

[24] Oda H, Yamamoto Y, Maeda K (2009). Neuropsychological profile of dementia with Lewy bodies. Psychogeriatrics.

[25] Ferman TJ, Smith GE, Kantarci K, Boeve BF, Pankratz VS, Dickson DW (2013). Nonamnestic mild cognitive impairment progresses to dementia with Lewy bodies. Neurology.

[26] Yoshizawa H, Vonsattel JP, Honig LS (2013). Early neuropsychological discriminants for Lewy body disease: an autopsy series. J Neurol Neurosurg Psychiatry.

[27] Petrova M, Mehrabian-Spasova S, Aarsland D, Raycheva M, Traykov L (2015). Clinical and neuropsychological differences between mild Parkinson’s disease dementia and dementia with Lewy bodies. Dement Geriatr Cogn Dis Extra.

[28] Petrova M, Pavlova R, Zhelev Y, Mehrabian S, Raycheva M, Traykov L (2016). Investigation of neuropsychological characteristics of very mild and mild dementia with Lewy bodies. J Clin Exp Neuropsychol.

[29] Petersen RC (2004). Mild cognitive impairment as a diagnostic entity. J Intern Med.

[30] American Psychiatric Association (2013). Diagnostic and statistical manual of mental disorders: DSM-5.

[31] Barberger-Gateau P, Commenges D, Gagnon M, Letenneur L, Sauvel C, Dartigues JF (1992). Instrumental activities of daily living as a screening tool for cognitive impairment and dementia in elderly community dwellers. J Am Geriatr Soc.

[32] Lawton MP, Brody EM (1969). Assessment of older people: self-maintaining and instrumental activities of daily living. Gerontologist.

[33] Israel L, Waintraub L (1986). Autonomie ou capacité fonctionnelle? Revue critique de quelques échelles actuellement utilisées en gériatrie pour l’évaluation des activités de la vie quotidienne. Psychol Med (Paris).

[34] Katz S, Ford AB, Moskowitz RW, Jackson BA, Jaffe MW (1963). Studies of the illness in the aged. The index of ADL: a standardized measure of biological and psychosocial function. JAMA..

[35] Scheltens P, Leys D, Barkhof F, Huglo D, Weinstein HC, Vermersch P (1992). Atrophy of medial temporal lobes on MRI in “probable” Alzheimer’s disease and normal ageing: diagnostic value and neuropsychological correlates. J Neurol Neurosurg Psychiatry.

[36] Dubois B, Feldman HH, Jacova C, DeKosky ST, Barberger-Gateau P, Cummings J (2007). Research criteria for the diagnosis of Alzheimer’s disease: revising the NINCDS-ADRDA criteria. Lancet Neurol.

[37] Lehmann S, Dumurgier J, Schraen S, Wallon D, Blanc F, Magnin E (2014). A diagnostic scale for Alzheimer’s disease based on cerebrospinal fluid biomarker profiles. Alzheimers Res Ther.

[38] Van der Linden M, Coyette F, Poitrenaud J, Kalafat M, Calicis F, Wyns C, Van der Linden M, Adam S, Agniel A, Baisset‐Mouly C (2004). L’épreuve de rappel libre/rappel indicé à 16 items (RL/RI‐16). L’évaluation des troubles de la mémoire. Présentation de quatre tests de mémoire épisodique (avec leur étalonnage).

[39] Barbeau E, Tramoni E, Joubert S, Mancini J, Ceccaldi M, Poncet M, Van der Linden M, Adam S, Agniel A, Baisset‐Mouly C (2004). Évaluation de la mémoire de reconnaissance visuelle: normalisation d’une nouvelle épreuve en choix forcé et utilité en neuropsychologie clinique. L’évaluation des troubles de la mémoire. Présentation de quatre tests de mémoire épisodique (avec leur étalonnage).

[40] Wechsler D (1997). Wechsler Adult Intelligence Scale—3rd Edition (WAIS-3).

[41] Dubois B, Slachevsky A, Litvan I, Pillon B (2000). The FAB: a Frontal Assessment Battery at bedside. Neurology.

[42] Tombaugh TN (2004). Trail Making Test A and B: normative data stratified by age and education. Arch Clin Neuropsychol.

[43] Cardebat D, Doyon B, Puel M, Goulet P, Joanette Y (1990). Formal and semantic lexical evocation in normal subjects: performance and dynamics of production as a function of sex, age and educational level. Acta Neurol Belg.

[44] Deloche G, Hannequin D (1997). Test de dénomination orale d’images – DO80.

[45] Rey A (1959). Test de copie et de reproduction de mémoire de figures géométriques complexes.

[46] Warrington EK, James M (1991). The Visual Object and Space Perception Battery.

[47] Mahieux-Laurent F, Fabre C, Galbrun E, Dubrulle A, Moroni C, groupe de réflexion sur les praxies du CMRR Île-de-France Sud (2009). Validation of a brief screening scale evaluating praxic abilities for use in memory clinics: evaluation in 419 controls, 127 mild cognitive impairment and 320 demented patients [in French]. Rev Neurol (Paris).

[48] Bertoux M, Funkiewiez A, O’Callaghan C, Dubois B, Hornberger M (2013). Sensitivity and specificity of ventromedial prefrontal cortex tests in behavioral variant frontotemporal dementia. Alzheimers Dement.

[49] Stone VE, Baron-Cohen S, Knight RT (1998). Frontal lobe contributions to theory of mind. J Cogn Neurosci.

[50] Ekman P, Friesen W (1976). Pictures of Facial Affect.

[51] Baron-Cohen S, Wheelwright S, Hill J, Raste Y, Plumb I (2001). The “Reading the Mind in the Eyes” Test revised version: a study with normal adults, and adults with Asperger syndrome or high-functioning autism. J Child Psychol Psychiatry.

[52] Metz-Lutz MN, Kremin H, Deloche G, Hannequin D, Ferrand I, Perrier D (1991). Standardisation d’un test de dénomination orale: contrôle des effets de l’âge, du sexe et du niveau de scolarité chez les sujets adultes normaux. Rev Neuropsychol.

[53] Mitolo M, Salmon DP, Gardini S, Galasko D, Grossi E, Caffarra P (2014). The new Qualitative Scoring MMSE Pentagon Test (QSPT) as a valid screening tool between autopsy-confirmed dementia with Lewy bodies and Alzheimer’s disease. J Alzheimers Dis.

[54] Li X, Rastogi P, Gibbons JA, Chaudhury S (2014). Visuo-cognitive skill deficits in Alzheimer’s disease and Lewy body disease: a comparative analysis. Ann Indian Acad Neurol.

[55] Nagahama Y, Okina T, Suzuki N (2015). Impaired imitation of gestures in mild dementia: comparison of dementia with Lewy bodies, Alzheimer’s disease and vascular dementia. J Neurol Neurosurg Psychiatry.

[56] Cormack F, Aarsland D, Ballard C, Tovee MJ (2014). Pentagon drawing and neuropsychological performance in dementia with Lewy bodies, Alzheimer’s disease, Parkinson’s disease and Parkinson’s disease with dementia. Int J Geriatr Psychiatry.

[57] Cagnin A, Bussè C, Gardini S, Jelcic N, Guzzo C, Gnoato F (2015). Clinical and cognitive phenotype of mild cognitive impairment evolving to dementia with Lewy bodies. Dement Geriatr Cogn Dis Extra.

[58] Pal A, Biswas A, Pandit A, Roy A, Guin D, Gangopadhyay G (2016). Study of visuospatial skill in patients with dementia. Ann Indian Acad Neurol.

[59] Ota K, Murayama N, Kasanuki K, Kondo D, Fujishiro H, Arai H (2015). Visuoperceptual assessments for differentiating dementia with Lewy bodies and Alzheimer’s disease: illusory contours and other neuropsychological examinations. Arch Clin Neuropsychol.

[60] Sahgal A, Galloway PH, McKeith IG, Lloyd S, Cook JH, Ferrier IN (1992). Matching-to-sample deficits in patients with senile dementias of the Alzheimer and Lewy body types. Arch Neurol.

[61] Mondon K, Gochard A, Marque A, Armand A, Beauchamp D, Prunier C (2007). Visual recognition memory differentiates dementia with Lewy bodies and Parkinson’s disease dementia. J Neurol Neurosurg Psychiatry.

[62] Philippi N, Noblet V, Duron E, Cretin B, Boully C, Wisniewski I (2016). Exploring anterograde memory: a volumetric MRI study in patients with mild cognitive impairment. Alzheimers Res Ther.

[63] Lambon-Ralph MA, Powell J, Howard D, Whitworth AB, Garrard P, Hodges JR (2001). Semantic memory is impaired in both dementia with Lewy bodies and dementia of Alzheimer’s type: a comparative neuropsychological study and literature review. J Neurol Neurosurg Psychiatry.

[64] Ferman TJ, Smith GE, Boeve BF, Graff-Radford NR, Lucas JA, Knopman DS (2006). Neuropsychological differentiation of dementia with Lewy bodies from normal aging and Alzheimer’s disease. Clin Neuropsychol.

[65] Troster AI, Fields JA, Testa JA, Paul RH, Blanco CR, Hames KA (1998). Cortical and subcortical influences on clustering and switching in the performance of verbal fluency tasks. Neuropsychologia.

[66] Troyer AK, Moscovitch M, Winocur G, Leach L, Freedman M (1998). Clustering and switching on verbal fluency tests in Alzheimer’s and Parkinson’s disease. J Int Neuropsychol Soc.

[67] Heitz C, Noblet V, Phillipps C, Cretin B, Vogt N, Philippi N (2016). Cognitive and affective theory of mind in dementia with Lewy bodies and Alzheimer’s disease. Alzheimers Res Ther.

[68] Aboulafia-Brakha T, Christe B, Martory MD, Annoni JM (2011). Theory of mind tasks and executive functions: a systematic review of group studies in neurology. J Neuropsychol.

[69] Ferman TJ, Smith GE, Boeve BF, Ivnik RJ, Petersen RC, Knopman D (2004). DLB fluctuations: specific features that reliably differentiate DLB from AD and normal aging. Neurology.

